# Stillbirth and infant death: mental health among low-income mothers in Mumbai

**DOI:** 10.1186/s12884-021-03754-0

**Published:** 2021-04-10

**Authors:** Lisa Roberts, Solomon J. Renati, Shreeletha Solomon, Susanne Montgomery

**Affiliations:** 1 School of Nursing, Loma Linda University, Loma Linda, USA; 2 Veer Wajekar A. S. & C. College, University of Mumbai, Mumbai, India; 3 Research Scholar, Martin Luther Christian University, Shillong, India; 4 School of Behavioral Health, Loma Linda University, Loma Linda, USA

**Keywords:** Stillbirth, Infant death, Perinatal grief, Postpartum depression, Mental health, Maternal health, India

## Abstract

**Background:**

India has the highest number of stillbirths and the highest neonatal death rate in the world. In the context of its pronatalist society, women who experience perinatal loss often encounter significant social repercussions on top of grief. Furthermore, even when pregnancy outcomes were favorable, adverse life circumstances put some women at risk for postnatal depression. Therefore, perinatal loss and postnatal depression take a heavy toll on women’s mental health. The purpose of this study is to assess mental health among a sample of Mumbai slum-dwelling women with a history of recent childbirth, stillbirth, or infant death, who are at risk for perinatal grief, postnatal depression, or mental health sequelae.

**Methods:**

We conducted a mixed method, cross-sectional study. A focus group discussion informed the development of a comprehensive survey using mainly internationally validated scales. After rigorous forward and back-translation, surveys were administered as face-to-face structured interviews due to low literacy and research naiveté among our respondents. Interviews were conducted by culturally, linguistically, gender-matched, trained research assistants.

**Results:**

Of our reproductive age (*N* = 260) participants, 105 had experienced stillbirth, 69 had a history of infant death, and 25 had experienced both types of loss. Nearly half of the sample met criteria for postnatal depression, and 20% of these women also met criteria for perinatal grief. Anxiety and depression varied by subgroup, and was highest among women desiring an intervention.

**Conclusions:**

Understanding factors contributing to women’s suffering related to reproductive challenges in this pronatalist context is critically important for women’s wellbeing.

## Background

Globally, stillbirth and infant death constituted nearly half of the under-5 mortality burden in 2015 [1, 2]. Currently, India’s under-5 mortality rate is estimated at 35.8 per 1000 live births, with a reported annualized 5.4% decrease from 2010 to 2019 [3]. Furthermore, estimates for stillbirth are 25.3 per 1000 births [4], neonatal deaths (within the first 28 days of life) 29.06 per 1000 live births, and post-neonatal death (> 28 days to 1 year of life) 11.74 per 1000 live births [5]. While declining stillbirth and infant death rates have been noted, rates are likely underestimated due to difficulty in capturing these data, including a reluctance to report such outcomes [6–8].

Known risk factors for stillbirth and infant death in low-and-middle-income countries (LMICs) include young or advanced maternal age, higher parity, lack of prenatal care, and malpresentaton [4, 9]. Perinatal loss and grief is a global phenomenon that affects mothers across cultures, including in India [10, 11] and results in mental health sequalae including complicated grief, anxiety, depression, posttraumatic stress, suicidal ideation, and marital disruption [12–14]. Moreover, in the context of a pronatalist society (promoting human reproduction) such as India, women who have a perinatal loss often experience stigma, guilt, blame, loss of status, discrimination, mistreatment, and abandonment or divorce on top of grief [13–15]. Perinatal loss, whether due to stillbirth or infant death, takes a heavy toll on women’s mental health [13, 16].

A high prevalence of depression in slums has been documented, with women at higher risk than men [17, 18]. Yet, among the already vulnerable slum dwelling women, the burden of perinatal grief goes unaddressed in the context of the profound mental health treatment gap in the country [18]. Compounding issues of longstanding stigmatization of mental health and the lack of mental health professionals or programming [19], provide these women few if any options to seek help. Even women who give birth to a healthy infant are at risk for postnatal depression. Risk of postnatal depression is increased in women with a history of mental health disorders; obstetric history (preterm birth, instrument assisted or cesarean delivery); young or advanced maternal age; lifestyle factors (poor nutrition, poor sleep patterns); as well as social factors including lack of social support, domestic violence, socioeconomic level, unemployment, low education, and low income [20–22]. The prevalence rate of postnatal depression among Indian women is estimated to range from 19 to 33.5%, and is likely undiagnosed and/or underreported, particularly among marginalized groups in low-resource settings [23]. Additionally, son preference, which is prevalent in Indian society, has been associated with perinatal depression [22, 24, 25].

With the importance placed on women’s childbearing in this traditionally patriarchal and pronatalist society, failure to produce offspring, or specifically a son, creates loss of status on top of grief, putting affected women at high risk for mental health problems. Therefore, the purpose of this study is to assess mental health among a sample of Mumbai slum dwelling women with a history of recent childbirth, stillbirth, or infant death, who are at risk for perinatal grief, postnatal depression, or mental health sequelae. Ultimately, better understanding of mental health needs, as well as inherent strengths, among these vulnerable women may inform appropriate policies and interventions to decrease their risks in the future.

## Methods

We conducted a mixed methods study during which we asked community health workers (CHWs) to be part of our formative work to guide a comprehensive survey pertaining to women’s reproductive problems and related psychosocial issues.

### Qualitative methods

A focus group of (*n* = 7) with CHWs was conducted in Marathi, a local language, audio-recorded, transcribed, and analyzed using standard methods [26]. The CHWs who participated in this focus group are well known to local women, as they are responsible for vital statistic reporting of all pregnancies and child births. They themselves generally reside in the communities they work in and are thus knowledgeable informants and key community stakeholders. We conducted the focus group encouraging open discussion among the participants, using a semi-structured guide with questions aligned with our theoretical model (social expectations for women of reproductive age, childbearing concerns and challenges/stress in the community).

### Setting

All data collection took place in a large slum in Mumbai, that was initially settled by migrant families who came to the city for work. It has become a permanent community with long-term resident families. Typical of most slums, this community lacks basic services such as safe drinking water, and sufficient electricity, sewage and waste disposal. While residents are hard-working, optimistic people, they are plagued by poverty and many other social determinants of health that negatively affect health outcomes. For example, stunting has been noted in 50% of the children in one area of the slum [27].

### Quantitative methods

We developed a survey aligned with identified themes, using validated scales commonly used in international settings, whenever possible. The survey included socio-demographic questions and reproductive history, as well as scales pertaining to social support, religious coping, coping style, autonomy, mental health, postnatal depression, and satisfaction with life. Additionally, women with a history of stillbirth completed the Perinatal Grief scale and more detailed questions pertaining to each stillbirth experienced, whereas women with a history of infant death completed more detailed questions pertaining to each infant lost.

Institutional Review Board (IRB) approval was received from both the US and Indian academic institutions of the authors. We translated all study materials into Hindi and Marathi, the local languages spoken by our target population. Bilingual scholars outside of the research team completed the translations utilizing the independent forward and backward translation technique to ensure cultural and functional equivalence, rather than simply literal translation [28].

Recruitment was guided by the CHWs, popularly known as Accredited Social Health Activist (ASHA) workers, for a purposive sample of women who had been pregnant within the last 12 months, regardless of pregnancy outcome. After verbally reviewing the informed consent form, participants signed or marked with their thumbprint, then surveys were conducted as face-to-face interviews (*N* = 260) due to low literacy levels and research naiveté of the target population. Culturally, linguistically, gender-matched, trained research assistants read the questions and response options verbatim to participants and recorded the responses. We conducted this assessment to explore the need for a future stress reduction intervention. For women who expressed an interest in a future intervention, we also collected contact information, which was accessible only to the researchers in a separate file from the de-identified survey data.

### Measures

Socio-demographic variables included age, marital status, religion, education, occupation, and socioeconomic status. Additional descriptive variables included general health status and reproductive history, as well as details pertaining to stillbirth/s or infant death/s.

### Validated scales

*The Hopkins Symptoms Check List – 10 (HSCL-10)* has been used successfully in a number of low income and low educational international settings including Pakistan (Urdu version), and Chhattisgarh, India (Hindi translation) where it was found to have good reliability, with a Cronbach’s alpha of 0.84 [29, 30]. It was therefore chosen for use for this population who shares some similarities. The measure consists of 10 items, which are rated on a Likert-type scale ranging from (1) not at all, to (4) extremely, with higher scores representing more symptoms of anxiety and depression. Like Syed et al. [30], we used a cut-off score, with a mean of 1.65 or greater indicating presence of notable mental health symptoms (anxiety and depression).

The 12-item version of *the Social Provision Scale (SPS)* assesses the perception of social support received from others [31]. A four-point Likert-type scale of (1) strongly disagree, to (4) strongly agree is summed for a possible score ranging from 12 to 48, with higher scores indicating greater social support perceived. This scale was previously used among poor women in rural Chhattisgarh, India and found to be reliable as indicated by Cronbach’s α of .74 [32].

The *short form of the Brief RCOPE* is a 7-item scale measuring positive and negative religious coping (three questions each) with response options (0) not at all, to (3) a great deal, and a final question measuring to what extent religion is used to cope with stressful situations. The Likert-type response options for the last item are (0) not involved at all, to (3) very involved [33, 34]. This scale too was previously found to be adequately reliable in a sample of poor Indian women [32].

*The Shortened Ways of Coping-Revised (SWC-R)* is a 14-item Likert-type scale containing two sub-scales, wishful thinking and practical coping, as two distinct coping strategies [35]. Each sub-scale is summed separately, with higher scores indicating more frequent use of the particular coping strategy. Response options are (0) never used, to (3) regularly used. Previously used in India, the SWC-R was found to be reliable [36].

*The Edinburgh Postnatal Depression Scale (EPDS)* is a 10-item scale with a summed score ranging from 0 to 30, with scores 10 or greater indicating possible depression. The last item may also indicate suicidal thoughts [37]. The EPDS has been used and validated in many languages [38] and among Indian women and similar populations [39].

*The Perinatal Grief Scale (PGS)* is a 33-item scale utilizing Likert-type responses of (1) strongly disagree to (5) strongly agree. Two items are reversed and then all items are summed for a possible score ranging from 33 to 165. Higher scores represent more intense grief, with a cut-off score of ≥91 indicating a high degree of grief. The PGS has been used in many countries, including India [40, 41].

*The Satisfaction with Life Scale (SWLS)* is a 5-item scale with response options (1) strongly disagree to (7) strongly agree. Items are summed for a possible score of five to 35, with higher scores indicating greater satisfaction with life [42]. The SWLS is easily understood and applicable in diverse populations and settings [29, 42]. Cronbach’s alphas on all validated scales were acceptable, ranging from .71 to .93.

### Additional scales

#### Social Norms

An 8-item author developed scale used in previously in India [36], measures social norms pertaining to reproductive expectations employed Likert-type response option (1) strongly disagree, to (5) strongly agree. Items were summed, for a possible score of 8–40, with higher scores indicating greater endorsement of social norms. Cronbach’s α = .51.

#### Autonomy

An author developed autonomy scale used in previous studies in India [36], contains questions specific to women in Indian society who often have low autonomy. This four-item scale is summed, so that higher scores indicate greater autonomy. Cronbach’s α = .70.

### Quantitative analysis methods

Descriptive analyses were conducted using frequencies for nominal/categorical variables and Mean with Standard Deviation for continuous demographic and reproductive history variables. Chi square and t-tests were used to determine significant difference between subgroups. Bi-variable associations with mental health (HSCL) and co-variates were conducted using Pearson’s correlations to inform multivariate analyses, for model building purposes. Linear regression then explored significant variables with mental health.

## Results

### Qualitative findings

Relevant findings, summarized in emerging themes from the focus group (FG) discussion include reproductive expectations, causes of stillbirth and infant death, family/community response to perinatal loss, and distress related to reproductive challenges.

**Theme 1: Reproductive Expectations.** The ASHA workers (*N* = 7) confirmed that early marriage is common, with most women about 18 years of age at the time of marriage in their communities. The predominant social expectation is that women are to begin having children soon after marriage, and take care of the household. As stated by one ASHA worker, *“Women are married into the household. Usually daughter in law works at home and mother in law goes out for work*.*”* Another stated “*Yes, whether she is a housewife or working outside she is expected to have a baby within a year of marriage.*” The rest of the group agreed, further reiterating the importance of having a baby.

**Theme 2: Causes of stillbirths.** When asked about stillbirths in the community the ASHA workers discussed what they thought might cause stillbirths to occur and how the community responds. Causes of stillbirth were primarily attributed to lack of prenatal care, fear of going to the hospital, under recognition of troubling signs and symptoms, and adherence to traditions rather than seeking or following medical advice.

“*In some cases, females do not go for regular check-up during their pregnancy period.*”

“At times, even after their delivery date has passed, ladies do not go to the doctor thinking that they are not having labor pain and they go only when the pain will start.”

“*Mother- in- laws discount the visit to doctors and hospital by saying in old times where was hospital and they all delivered so many children [at home].”*

Causes of infant death were characterized in terms of infectious disease (fever, vomiting, diarrhea) related to a lack of hygiene, congenital defects, and unknown factors.

“Some babies are born with heart problems which is not revealed by the doctors during sonography.”

“Many times, doctors don’t explain the causes/reasons for infant death.”

**Theme 3: Family/community response to perinatal loss.** Family and community responses were characterized as providing emotional support, but at the same time the mother being pressured to conceive again soon.

“It is also observed that immediately after the loss of baby, the female is seen to be pregnant with another baby within couple of months. Even though a gap of one year is advised, others don’t agree and they want the women to be pregnant fast.”

“They do not follow any family planning. No gap is ensured in between births.”

**Theme 4: Distress related to reproductive challenges.** The reproductive expectations and social consequences (demoted to lower status within the household, blame, etc.) were noted to add to women’s distress after perinatal loss. The ASHA workers also indicated that unfortunately blaming of the mother for the death does occur. While some women experiencing perinatal loss are “not tortured much,” others endure significant emotional distress due to family/community response “*Females are blamed for the death of the baby or stillborn.”* Furthermore, the participants described social consequences of perinatal loss, including lower social status, displacement, abuse, divorce, or abandonment.

Participants described minimal rituals after infant death or stillbirth, stating that the body is given to the father for burial. The mother is often not allowed to see or touch the baby. Grief and distress were described as affecting the daily lives of the mothers emotionally and mentally. As one participant put it, “*Women are afraid that what has happened before will happen again during the next pregnancy.”* Other ASHA workers chimed in describing the distress they have observed, including grief, fear, crying, intense distress, withdrawal, isolation, and emotional trauma. Participants discussed the intensity and length of grief as variable but often severe when it occurred; they also indicated that if a wellness and resilience program were offered to help address this, the women would attend.

“In case of stillbirth or death of baby within a year of birth the females become reserved, they talk very less, avoid crowd or communications. Her behavior changes, she avoids talking or she talks less with people around her.”

“The baby died in the womb in the 9^th^ month and the mother’s condition was very bad. Women suffered from severe emotional trauma.”

These four themes (reproductive expectations, causes of stillbirth/infant death, family/community response to perinatal loss, distress related to reproductive challenges) are naturally somewhat overlapping and compounding. FG participants described a pattern of emotional distress and behaviors after perinatal loss, which can be identified as mental health sequelae that they felt was compounded by the societal and family reaction to the loss.

### Quantitative results

#### Participants

The study sample (*N* = 260) consisted of women 18 to 42 years old, residing in established slums of Mumbai. Given our study interest we oversampled women with a history of reproductive challenges. More than half (56.4%) live in a joint family context rather than a nuclear family (43.6%), mostly identifying as a wife or daughter-in-law (98.4%), and had been married for an average of 8.23 years (*SD* 5.61). The participants were primarily Hindu (48.1%) and Muslim (40.4%), but also include Buddhists (9.2%), or other religions (2.7%) such as Christians and Banjaris. Nearly half (46%) were illiterate or had only primary level education, and 88.1% worked as unskilled workers or homemakers. In general, they deemed themselves as physically and mentally healthy (70.4 and 78.3% indicating no problems respectively), and most (77.4%) did not use any contraceptive method (see Table 1 for further details).

**Table 1:** Demographics of All Participants (*N* = 260)

Characteristic	All Participants
	Frequency ***n*** (%)
**Household position (*n*** **= 260)**	
Wife	154 (59.2)
Daughter-in-law	102 (39.2)
Head of household	2 (0.8)
Daughter	2 (0.8)
**Religion (*n*** **= 260)**	
Hindu	125 (48.1)
Muslim	105 (40.4)
Buddhist	24 (9.2)
Other^a^	6 (2.3)
**Highest level of education (*n*** **= 260)**	
None (illiterate)	41 (15.8)
Primary	94 (36.2)
Secondary	80 (30.8)
Higher-secondary or graduate	45 (17.3)
**Current occupation (*n*** **= 260)**	
Unskilled worker/Homemaker	229 (88.1)
Semi-skilled worker to semi-professional	31 (11.9)
**Monthly family income (*n*** **= 256)**	
≤ Rs. 2091–10,356	99 (38.7)
Rs. 10,357–20,714	140 (54.7)
Rs. 20,715–41,430+	17 (6.6)
**Time to reach a health facility (*n*** **= 260)**	
Less than 15 min	105 (40.4)
15 to 30 min	151 (58.1)
More than 30 min	4 (1.5)
**Tobacco/paan use (*n*** **= 260)**	
None	231 (88.8)
Tobacco/paan use	29 (11.2)
**Health problems (*n*** **= 257)**	
None	183 (70.4)
Anemia	56 (21.5)
Other^b^	21 (8.1)
**Psychosocial factors (*n*** **= 258)**	
None	202 (78.3)
Anxiety	52 (20.2)
Depression	21 (8.1)
Domestic violence	11 (4.2)
**Family style (*n*** **= 259)**	
Nuclear	113 (43.6)
Joint	146 (56.4)
**Birth intervals (*n*** **= 184)**	
< 2 years	78 (42.4)
> 2 years	106 (57.6)
**Contraceptive method used (*n*** **= 257)**	
None	199 (77.4)
Sterilization	21 (8.2)
Other^c^	37 (14.4)
	***M*** **(SD)**
**Age (*n*** **= 258)**	26.26 (4.83)
**Years married (*n*** **= 260)**	8.23 (5.61)
**Mother’s age at first delivery (*n*** **= 257)**	20.11 (3.00)

Other^a^ = Christian, Banjari

Other^b^ = Thyroid disease, weakness, malaria, hypertension, tuberculosis, kidney stones, calcium deficiency

Other^c^ = Medicine, condoms, IUD, surgery

Women with stillbirth loss (*n* = 105) had experienced 1–5 stillbirths (*M* = 1.45, *SD* 0.83 stillbirths) and were significantly more likely to have psychosocial factors than the rest of the sample. Significantly more of these women identified as Hindu, reported working as semi-skilled or semi-professionals, and reported living in nuclear families compared to the other participants. Additionally, on average, women with stillbirth loss, compared to other participating women, were significantly older, had been married longer, and had a higher number of pregnancies (Table 2 provides further details).

**Table 2:** Significant demographics differences between women with stillbirth compared to others

Characteristic		Without Stillbirth Loss ***n*** = 153	With History of Stillbirth only ***n*** = 105
		Frequency ***n*** (%)	Frequency ***n*** (%)
Religion* **(*n*** **= 260)**	Hindu	72 (46.5)	53 (50.5)
	Muslim	70 (45.2)	35 (33.3)
	Buddhist	10 (6.5)	14 (13.3)
	Other^a^	3 (1.9)	3 (2.9)
Current occupation* (*n* **= 260)**	Unskilled worker/Homemaker	142 (91.6)	87 (82.9)
	Semi-skilled worker to semi-professional	13 (8.4)	18 (17.1)
Psychosocial factors* (*n* **= 258)**	None	132 (85.2)	70 (68.0)
	Anxiety	22 (14.2)	30 (29.1)
	Depression	1 (0.6)	1 (1.0)
	Domestic violence	0	2 (1.9)
Family style* (*n* **= 259)**	Nuclear	57 (36.8)	56 (53.8)
	Joint	98 (63.2)	48 (46.2)
		***M*** **(SD)**	***M*** **(SD)**
Years of age*** (*n* **= 258)**		24.95 (4.34)	28.16 (4.90)
Years married*** (*n* **= 260)**		6.7 (4.94)	10.47 (5.82)
Number of pregnancies*** (*n* **= 260)**	(ranged from 1 to 11)	2.46 (1.50)	3.90 (1.70)

Note: * = *p* < .05, *** = *p* < .001

Other^a^ = Christian, Banjari

Women with a history of infant death (*n* = 69) had lived through this experience 1–4 times (*M* = 1.29, *SD* 0.60 infant deaths). They were also significantly different to the rest of the participants: most of them identified themselves as a wife rather than a daughter-in-law, they were more likely to be semi-skilled or semi-professionals, more often reported that they had no health problems, but also had a higher percentage of health problems other than anemia. These women were significantly older, had been married longer, and had a history of more pregnancies than the rest of the participants, as detailed in Table 3.

**Table 3:** Significant demographics differences between women who had experienced infant death compared to others

Characteristic		Without Infant Death Loss ***n*** = 191	With Infant Death Loss Only ***n*** = 69
		Frequency ***n*** (%)	Frequency ***n*** (%)
Household position* (*n* **= 260)**	Wife	103 (53.9)	51 (73.9)
	Daughter-in-law	84 (44.0)	18 (26.1)
	Head of household	2 (1.0)	0
	Daughter	2 (1.0)	0
Current occupation* (*n* **= 260)**	Unskilled worker/Homemaker	173 (90.6)	56 (81.2)
	Semi-skilled worker to semi-professional	18 (9.4)	13 (18.8)
Health problems* (*n* **= 260)**	None	133 (69.6)	50 (72.5)
	Anemia	48 (25.1)	8 (11.6)
	Other^a^	10 (5.2)	11 (15.9)
		***M*** **(SD)**	***M*** **(SD)**
Years of age*** (*n* **= 258)**		25.37 (4.53)	28.72 (4.82)
Years married*** (*n* **= 260)**		7.13 (5.26)	11/.26 (5.47)
Number of pregnancies*** (*n* **= 260)**	(ranged from 2 to 8)	2.72 (1.64)	3.94 (1.68)

Note: * = *p* < .05, *** = *p* < .001

Other^a^ = Thyroid disease, weakness, malaria, hypertension, tuberculosis, kidney stones, calcium deficiency

Some of the women had experienced both stillbirth and infant death (*n* = 25). These women were significantly more likely to have secondary or higher education levels (52% vs. 47.7%), were older (*M* = 30.20, *SD* 5.47 vs. *M* = 25.83 *SD* 4.57), had been married longer (*M* = 13.28, *SD* 5.54 vs. *M* = 7.69, *SD* 5.36), and had more pregnancies (*M* = 4.56, *SD* 1.42 vs. *M* = 2.89, *SD* 1.69) compared to the rest of the sample (range 2–8).

When comparing women who had expressed an interest in a future intervention (*n* = 74) with those who did not (*n* = 180), we found no significant differences in demographics or reproductive history except for occupation; *X*^2^ (1) = 12.03, *p* = .001, with a higher percentage of semi-skilled/semi-professional workers among women wanting the intervention. Additionally, self-assessment of general health status and psychosocial health differed. This subgroup was statistically more likely to self-identify as having general health problems *X*^2^ (2) = 7.55, *p* = .021, and psychosocial factors *X*^2^ (31) = 16.46, *p* = .000.

### Variables of interest

Overall participants in our sample reported in the mid-range of social support provided (*M* 38.84, *SD* 5.94), higher use of negative rather than positive religious coping (*M* = 8.74, *SD* 2.16 vs *M* = 4.36, *SD* 2.40), employed both practical and emotion-based wishful thinking as coping strategies (*M* = 14.05, *SD* 4.16 and M = 10.51, SD 4.19 respectively), identified their level of autonomy as slightly above the mid-range (*M* = 7.49, *SD* 2.02), and had average satisfaction with life (26.11; *SD* 7.29).

Our indicator for mental health (HSCL) was on average slightly above the 1.65 cut-off score (*M* = 1.68, *SD* 0.72). The average EPDS score of 9.35 (*SD* 6.35) was below the cut-off score of ≥10 used to indicate postnatal depression, of note however, 119 participants had a score ≥ 10. Among these 119 women (45% of the total sample), the EPDS average was 14.75 (*SD* 4.27). Nearly all of these women had experienced either stillbirth (*n* = 58, 49%), infant death (*n* = 34, 29%), or both types of loss (*n* = 14, 12%). Among women with a history of stillbirth (*n* = 105) the perinatal grief score was below the cut off score of 91 with a mean of 78.75 (*SD* 26.97). However, 21 of these women (20% of the subgroup of participants with a history of stillbirth) had perinatal grief scores above the cut off (*M* = 113.85, *SD* 12.60).

### Independent samples *t*-tests

We used independent samples t-tests to analyze differences between subgroups of the study sample regarding mental health (HSCL) and other scale variables.

Women with a history of stillbirth were significantly higher in their perception of social provision of support received *t* (249) = − 2.06, *p* = .041, 95% CI [− 3.21, − 0.07]; more likely to employ wishful thinking as a coping strategy *t* (255) = − 3.28, *p* = .001, 95% CI [− 2.74, − 0.68], and had higher postnatal depression scores *t* (246) = − 1.97, *p* = .047, 95% CI [− 3.25, − 0.02] compared to women without a history of stillbirth.

Women who had experienced infant loss perceived significantly more social support *t* (249) = − 2.08, *p* = .039, 95% CI [− 3.42, − 0.09], positive religious coping *t* (256) = 2.23, *p* = .027, 95% CI [0.07, 1.23], and higher levels of autonomy *t* (250) = − 2.10, *p* = .037, 95% CI [− 1.15, − 0.03] compared to women who had not experienced infant death.

Among women who desired a future intervention, HSCL was significantly higher and above the 1.65 cut-off score (*M* = 2.08, *SD* 0.87). They employed more wishful thinking, had greater autonomy, higher EPDS scores which on average were above 10 indicating postnatal depression (*M* = 12.35, *SD* 7.60), greater perinatal grief which exceeded the cut-off score of 91 (*M* = 98.87, *SD* 24.70), perceived less social support, and had lower life satisfaction (see Table 4 for details).

**Table 4:** Comparing women who expressed interest in an intervention with all others using independent samples *t*-tests

	Women with expressed interest in intervention ***n*** = 74	All other women ***n*** = 186			
Parameter	***M (SD)***	***M (SD)***	***t*** (256)	***p***	95% CI
HSCL	2.08 (0.87)	1.53 (0.56)	−5.915	.000	[−0.74–0.36]
Wishful thinking	11.60 (4.39)	10.07 (4.03)	−1.542	.007	[−2.67, −0.41]
Autonomy	7.92 (1.94)	7.33 (2.02)	−0.60	.036	[−1.14, −0.39]
EPDS	12.35 (7.06)	8.19 (5.66)	−4.16	.000	[−5.85, −2.46]
Perinatal grief	98.87 (24.70)	68.89 (22.31)	−29.97	.000	[−41.45, −18.50]
Social support	36.70 (6.80)	38.98 (5.46)	2.279	.006	[0.65, 3.89]
Life satisfaction	22.92 (9.04)	27.40 (6.03)	4.483	.000	[2.20, 6.74]

### Bivariate analysis

For model building purposes we used bivariate analysis to explore if any of the independent variables were significantly associated with mental health (HSCL). Significantly associated variables included lower socioeconomic status (SES) (−.251, *p* = .000), poorer general health (.217, *p* = .001), more self-identified psychosocial factors (.420, *p* = .000), higher number of stillbirths (.135, *p* = .032), higher postnatal depression (.773, *p* = .000), higher perinatal grief (.714, *p* = .000), less social support (−.350, *p* = .000), more positive and negative religious coping (.162, *p* = .010 and .276, *p* = .000 respectively), more use of wishful thinking as a coping strategy (.343, *p* = .000), and lower life satisfaction (−.547, *p* = .000). When only women who expressed an interest in intervention (*n* = 74) were selected, the same variables were significant in our bivariate analysis.

### Analysis of predictors of mental health

Bi-variably significantly associated variables were used to conduct multivariate analysis with mental health (HSCL). These included SES, general health, psychosocial factors, stillbirths, wishful thinking, postnatal depression, perinatal grief, social support, positive and negative religious coping, and life satisfaction. Missing data was evaluated and revealed 12.7% missing data for the SES variables. Therefore, multiple imputation was performed prior to the regression analysis. In the first model, comparative socioeconomic status, general health, postnatal depression, and perinatal grief remained significant, explaining 67% of the variance.

To explore a more parsimonious model, only significant variables of the first model were entered in the second analyses, and all four variables remained significant, but the variance decreased to 63%. See Table 5 for a summary of the regression analyses.

**Table 5:** Summary of Regression Analysis for Variables Predicting Mental Health (HSCL) among women with a history of loss (*n* = 149)

Model 1: All significant bivariables of HSCL included			
Variable	*B*	*SE B*	β
Constant	1.48	0.33	
Comparative SES	−0.12	0.05	−.12*
General health	0.13	0.05	.13*
Psychosocial problems	0.14	0.07	.11
Number of stillbirths	0.03	0.03	.06
Coping style- Wishful thinking	0.01	0.01`	.05
Postpartum depression	0.05	0.01	.43***
Perinatal grief	0.01	0.01	.20**
Social support	−0.01	0.01	−.10
Positive religious coping	−0.01	0.02	−.01
Negative religious coping	0.01	0.02	.04
Life satisfaction	−0.01	0.01	−.12
*R*^2^	.67		
*F* for change in *R*^2^	24.35***		

*p < .05, **p < .01, ***p < .001 Note: Loss was either due to stillbirth or infant death

## Discussion

This paper examined mental health among Mumbai slum-dwelling women who had experienced stillbirth or infant death, using mixed-methods. The sample represents a vulnerable population on many levels (e.g. poverty, gender, and low status within the family/community hierarchy) who are at risk for mental health sequelae after perinatal loss. As noted in our qualitative findings, their lives are clearly affected by societal reproductive expectations, which is consistent with the literature indicating that becoming a mother is culturally anticipated within the first year of marriage, and in fact, women who do not conceive face considerable social pressure and coercion [43]. The context in which stillbirth or infant death occurs, and family or community responses to perinatal loss, as noted in our qualitative findings are also consistent with the literature. The distress described by FG participants paints a vivid picture of the mental health sequelae that can occur after women experience stillbirth or infant death. However, maternal mental health has not received much consideration in India’s national mental health program [21].

Yet overall, mental health symptomology as indicated by HSCL was not as high as expected among these low-income women living in established Mumbai slums who have reproductive concerns. Most were married at about 18 years of age, and had been married long enough to have established families. The lack of contraceptive use likely reflects the pronatalist culture, but may also represent unmet reproductive health needs which was estimated to be 11.7% in Maharashtra for 2015 and expected to remain at about 12% through 2030 [44]. While this is represents a lower percentage of unmet need for modern contraception than some other states [44], averages hide variances across subgroups within states. Therefore, the unmet need may be much higher among low-income women. However, in this sample of women, a higher total number of pregnancies among women with a history of loss indicates that they have kept trying to get pregnant, hoping for better outcomes. Not only does this reflect the pronatalist society, but also a desire to maintain or gain a higher social status and/or prevent untoward events associated with a lack of successful childbearing [13–15, 41, 43].

Despite these better than expected overall mental health outcomes, nearly half the sample screened positive for postnatal depression, and all but 13 of these women had experienced losses—stillbirths, infant deaths, or both. Poor maternal health and nutrition, sanitation issues, and delays in receiving health care or referral to specialty care after reaching a health care facility contribute to stillbirth and infant death prevalence in Mumbai slums [45].

Of note, women who had experienced loss perceived significantly more social support provided than the rest of the women. Their perceptions align with our observations of the social fabric of collectivistic life of slum residents. They seem to share each other’s joys and burdens, which aligns with the literature reporting that these are tight-knit communities that draw strength from each other [17]. Indeed, an unsolicited comment from one participant was “If I have to take many births (reincarnation), I would like to be reborn in this same community” indicating the value of the social support experienced. This aligns with Dasgupta et al. (2013) findings that persons living in low-income, urban housing settlements (slums) are afforded some buffering effect of a close-knit community of family, relatives, friends and neighborhood to help offset the woes of poor living conditions [46]. This inherent strength the women derive from the supports of their close-knit community also represents an opportunity when considering future interventions. Clearly future interventions should build on this inherent resiliency to support women in their journey of loss. Additionally, these women displayed self-insight in terms of need, and a willingness to engage in potential help. Those who had poorer mental health self-selected themselves as interested in further supports provided by a future intervention. These additional strengths in our participants are similar to the positive attitudes and willingness to learn noted in slum-dwelling mothers in another study [27].

However, the women’s experiences of loss, their dissatisfaction with social and medical services, and the discrimination slum residents face has a negative effect on mental health [17]. Women with a history of stillbirth were significantly more likely to employ emotion-based, wishful thinking as a coping strategy, whereas women who had lost an infant relied more on religious coping, compared to the larger sample. These findings are consistent with other studies [11, 47], and may be particularly so in a pronatalist context, which fosters wishing for another pregnancy and perhaps praying for another child.

The bivariable associations that were significantly associated with anxiety and depression symptomology (per HSCL) included participants’ status (self-evaluation of comparative SES, general health, psychosocial factors), number of stillbirths experienced, and self-report via validated measures (social support received, religious coping, life satisfaction, wishful thinking, postnatal depression, perinatal grief). When analyzed by logistic regression, these variables explained 67% of the variance in HSCL among women who had experienced loss. Comparative SES, general health, postpartum depression, and perinatal grief remained significant, indicating that they are important predictors of mental health.

The anguish following a loss may last months or years, leading to untoward mental health outcomes associated with bereavement. Mental health sequelae include complicated grief, depression, anxiety and tension, insomnia, somatic symptoms, suicidal inclinations social dysfunction and secondary social consequences [48]. Of note, the 74 women who expressed interest in an intervention, were indeed the very women who need it. This subgroup of women had the most depression and anxiety symptomology as indicated by having the highest HSCL mean, and also had postnatal depression and perinatal grief means well above the respective cut-off scores. Most bereaved people do recover over time, and are resilient (able to adapt) with time [48], however, some may need assistance to increase their resilience and wellbeing.

Results must be understood in light of some limitations. Purposive sampling, while appropriate to gain information related to loss experiences, limits generalizability. Additionally, self-reported measures are subject error in recall and response bias despite the use of tools that were validated for the Indian context. The cross-sectional design prevents causal conclusions, but our team included local experts, CHWs, who are part of the communities. With them as part of our team, our internationally experienced researchers discussed and attempted to contextualize findings, strengthening our conclusions.

Our study adds to the limited literature on the mental health sequalae of reproductive challenges by representing the experiences of vulnerable women who are seldom included. Between the near absolute lack of availability of any type of treatment options and the stigma associated with mental health care [18, 19], these women, who include many in need of mental health care, are the least likely to receive it. There is evidence that community mental health awareness programs have made some impact on stigmatization, however, traditional beliefs still deter use of allopathic treatment. Additionally, the combination of low public expenditure for mental health care and the growing private sector of expensive specialists, continues to keep mental health care out of reach for low-income populations in India [49]. Given the needs we identified, culturally tailored solutions to address these needs are called for, and future research pertaining to effective, low-cost community-based interventions are recommended.

## Conclusions

In a pronatalist context, women who have reproductive challenges are susceptible to mental health and social consequences. We found, that in our sample of low-income, slum dwelling women, the subgroup of women who expressed an interest in a future intervention were the women with highest need. Understanding the most important issues related to their suffering, as well as the inherent strengths in their communities, is the first step toward supporting their healing.

## Data Availability

The datasets used and analyzed during the current study are available from the corresponding author on reasonable request.

## Abbreviations

LMIC: Low-and-middle-income countries
CHW: Community health worker
IRB: Institutional review board
ASHA: Accredited Social Health Activist
HSCL: Hopkins Symptoms Check List
SPS: Social Provision Scale
RCOPE: Religious coping
SWC-R: Shortened Ways of Coping- Revised
EPDS: Edinburgh Postnatal Depression Scale
PGS: Perinatal Grief Scale
SWLS: Satisfaction with Life Scale
SES: Socioeconomic status
